# Supplementary material for: Optimised insert design for improved single-molecule imaging and quantification through CRISPR-Cas9 mediated knock-in
Source: Sci Rep. 2019 Oct 2;9:14219. doi: 10.1038/s41598-019-50733-9 (PMC6775134; doi:10.1038/s41598-019-50733-9)
Supplement: Supplementary file 1 — Supplementary Information [file 41598_2019_50733_MOESM1_ESM.pdf]

Optimised insert design for improved single-molecule imaging and quantification through CRISPR-Cas9 mediated knock-in

Abdullah O. Khan *[1], Carl W. White[2,3,4,5], Jeremy A. Pike[1,2], Jack Yule[2], Alexandre Slater[1], Stephen J. Hill[2,3], Natalie S. Poulter[1,2], Steven G. Thomas *[1,2], and Neil V. Morgan *[1]

[1]Institute of Cardiovascular Sciences, College of Medical and Dental Sciences, University of Birmingham, Edgbaston, B15 2TT
[2]Centre of Membrane and Protein and Receptors (COMPARE), University of Birmingham and University of Nottingham, Midlands, UK
[3]Division of Physiology, Pharmacology and Neuroscience, School of Life Sciences, University of Nottingham, Nottingham
[4]Molecular Endocrinology and Pharmacology, Harry Perkins Institute of Medical Research, Nedlands, WA, Australia
[5]Centre for Medical Research, The University of Western Australia, Crawley, WA, Australia

A

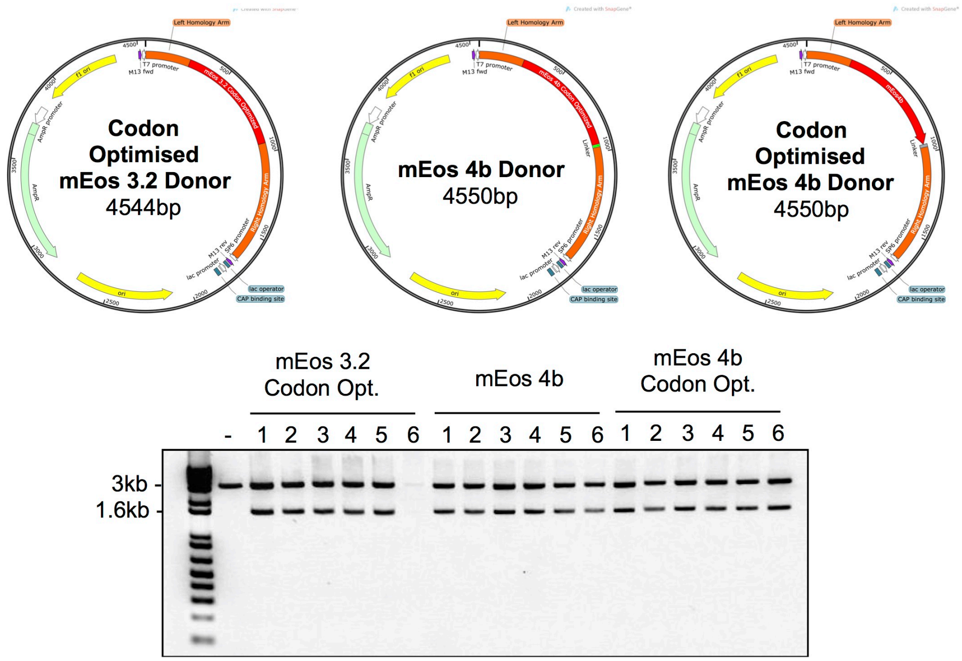


```
mEos 3.2                 -MSAIKPDMKIKLRMEGNVNGHHFVIDGDGTGKPFEGKQSMDLEVKEGGPLPFAFDILTT
mEos 3.2 Codon Opt.      -MSAIKPDMKIKLRMEGNVNGHHFVIDGDGTGKPFEGKQSMDLEVKEGGPLPFAFDILTT
mEos 4b                  MVSAIKPDMRIKLRMEGNVNGHHFVIDGDGTGKPYEGKQTMDLEVKEGGPLPFAFDILTT
mEos 4b Codon Opt.       MVSAIKPDMRIKLRMEGNVNGHHFVIDGDGTGKPYEGKQTMDLEVKEGGPLPFAFDILTT
                          :*******.************************:****:********************

                         AFHYGNRVFAKYPDNIQDYFKQSFPKGYSWERSLTFEDGGICNARNDITMEGDTFYNKVR
                         AFHYGNRVFAKYPDNIQDYFKQSFPKGYSWERSLTFEDGGICNARNDITMEGDTFYNKVR
                         AFHYGNRVFVKYPDNIQDYFKQSFPKGYSWERSLTFEDGGICNARNDITMEGDTFYNKVR
                         AFHYGNRVFVKYPDNIQDYFKQSFPKGYSWERSLTFEDGGICNARNDITMEGDTFYNKVR
                         *********.**************************************************

                         FYGTNFPANGPVMQKKTLKWEPSTEKMYVRDGVLTGDIEMALLLEGNAHYRCDFRTTYKA
                         FYGTNFPANGPVMQKKTLKWEPSTEKMYVRDGVLTGDIEMALLLEGNAHYRCDFRTTYKA
                         FYGTNFPANGPVMQKKTLKWEPSTEKMYVRDGVLTGDIEMALLLEGNAHYRCDFRTTYKA
                         FYGTNFPANGPVMQKKTLKWEPSTEKMYVRDGVLTGDIEMALLLEGNAHYRCDFRTTYKA
                         ************************************************************

                         KEKGVKLPGAHFVDHCIEILSHDKDYNKVKLYEHAVAHSGLPDNARR
                         KEKGVKLPGAHFVDHCIEILSHDKDYNKVKLYEHAVAHSGLPDNARR
                         KEKGVKLPGAHFVDHAIEILSHDKDYNKVKLYEHAVAHSGLPDNARR
                         KEKGVKLPGAHFVDHAIEILSHDKDYNKVKLYEHAVAHSGLPDNARR
                         ***************.*******************************
```

Fig. S1. Generation of mEos variant *TubA1B* donors. (A) Donor plasmids with identical linkers and homology arms harbouring mEos variants were designed. These include a codon optimised mEos 3.2 sequence, as well as a truly monomeric version of the protein reported as mEos 4b, and a respective codon optimised variant. (B) gBlocks were cloned into pGem-T-Easy backbones and validated through EcoRI digest. Negative controls are 3000bp in length, while successfully cloned vectors demonstrate an insertion which runs at approximagely 1600bp. Finally sequence alignments show differences in the 3.2 and 4b protein sequence, and that codon optimisation does not alter the reading frame or coding sequence reported. Amino acids marked in red are where the mEos 4b protein differs from its predecessor mEos 3.2.

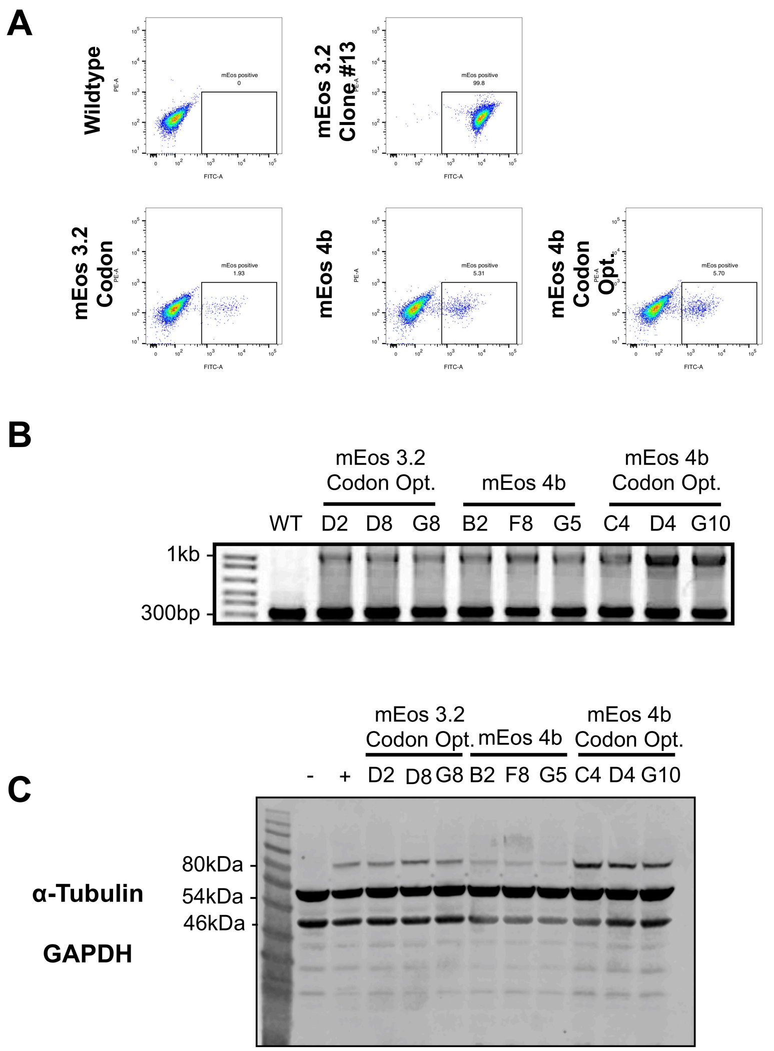

Fig. S2. Generation, sorting, and genomic validation of Hel 92.1.7 *TubA1B* CRISPR clones. (A) Hel 92.1.7 cells co-transfected with a guide targeting *TubA1B* and donor templates carrying codon optimised mEos 3.2, mEos 4b, and codon optimised mEos 4b were single cell sorted into a 96 well plate. (B) Clones were expanded and validated by PCR. The amplification of wild type DNA results in a 300bp band, while heterozygous clones positive for fluorophore insertion at the *TubA1B* locus also feature a second band at approximately 1000bp. (C) Full unedited western blot of gels presented in figure 2.

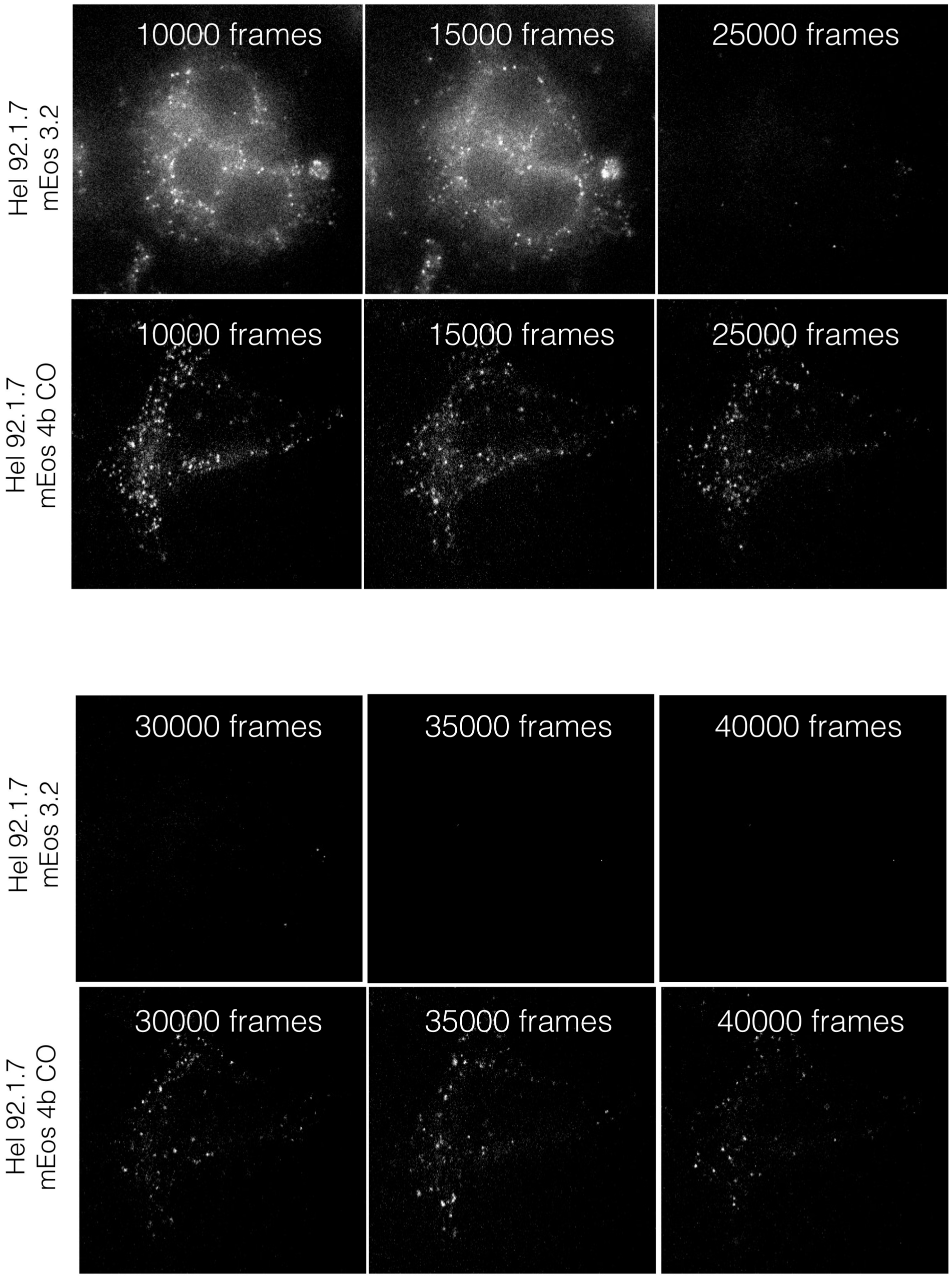

Fig. S3. Detections per frame in CRISPR knock-in mEos 3.2 and mEos 4b CO samples. While mEos 3.2 labelled cells rapidly bleach (by 25,000 frames), the higher expression evident in mEos 4b CO knock-in clones results in continued blinking and detection for up to 40,000 frames.

qRT-PCR Primers:

mEGFP Fwd:TAAACGGCCACAAGTTCAGC

mEos 3.2 Fwd: GACAATGCCAGACGATCCGGACTCAG

mEos 3.2 Codon Opt Fwd: CTGACAATGCGCGGCGATCCGGACTCAG

mEos 4b Fwd: GACAATGCCAGACGATCCGGACTCAG

mEos 4b Codon Opt Fwd: GATAACGCAAGACGATCCGGACTCAG

TUBA1B Reverse: GTGCACTGGTCAGCCAGCTTGCGAAT

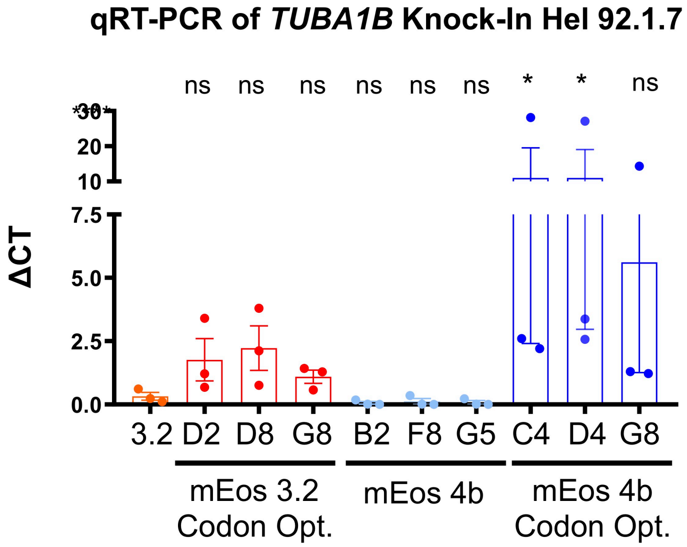

Fig. S4. qRT-PCR of *TUBA1B* knock-in clones supports Western blot data. Primers were designed to amplify correctly inserted donor sequences targeted by CRISPR to the *TUBA1B* locus. This data shows a significant increase in expression in 2 mEos 4b codon optimised clones (C4 and D4, * p = 0.0486, * p = 0.0488) when compared to a previously published mEos 3.2 clone. n = 3, One way ANOVA with multiple comparisons. S.D.

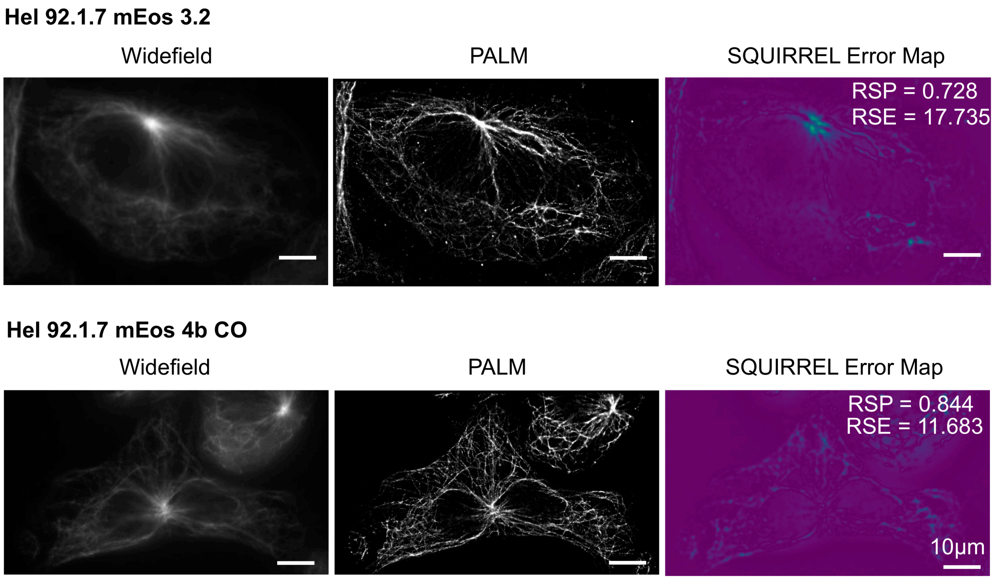

Fig. S5. Assessment of image quality using NanoJ Squirrel. PALM images of mEos 3.2 and mEos 4b CO knock-in clones were assessed for error as a measure of image quality. An improvement in RSP (global Pearson correlation co-efficient 0.728 and 0.844 respectively) and RSE (Resolution Scaled Error 17.735 and 11.683 respectively) was observed between the two clones.

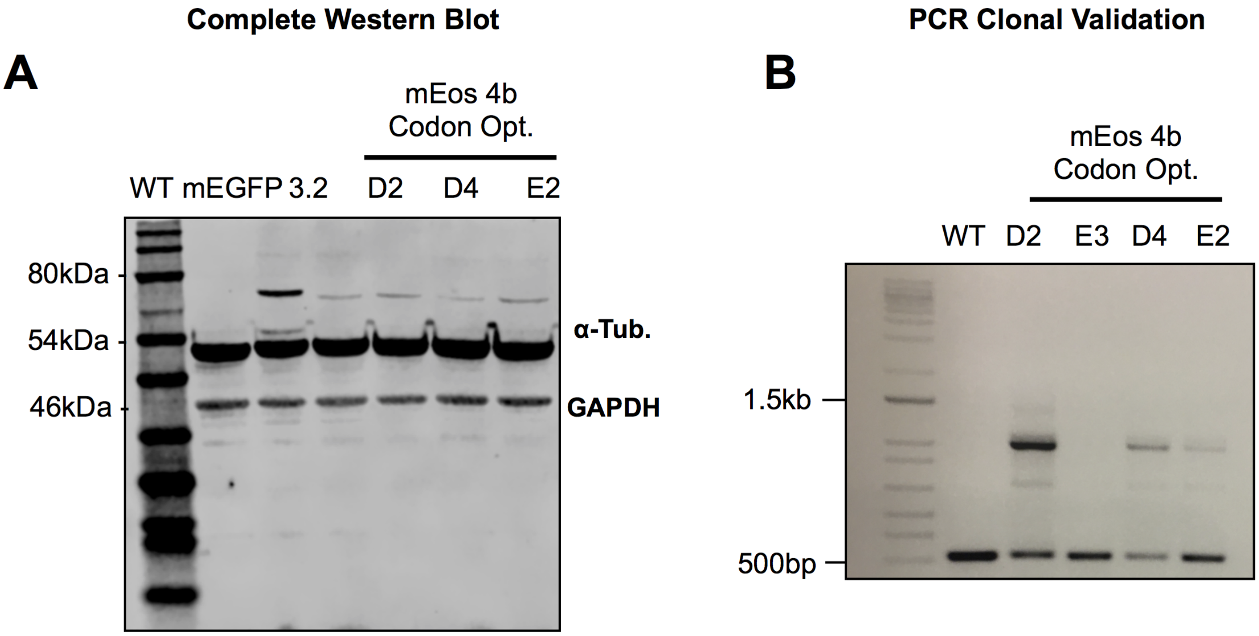

Fig. S6. Complete gels from main text. (A) Full gel of Hek293T *TubA1B* clones reported in figure 2. (B) PCR validation of mEos 4bCO clones, compared to wild type, clones D2, D4, and E2 demonstrate a band approximately 700 base pairs heavier than the wild type sequence, indicating a successful heterozygous insertion of the sequence at this locus.

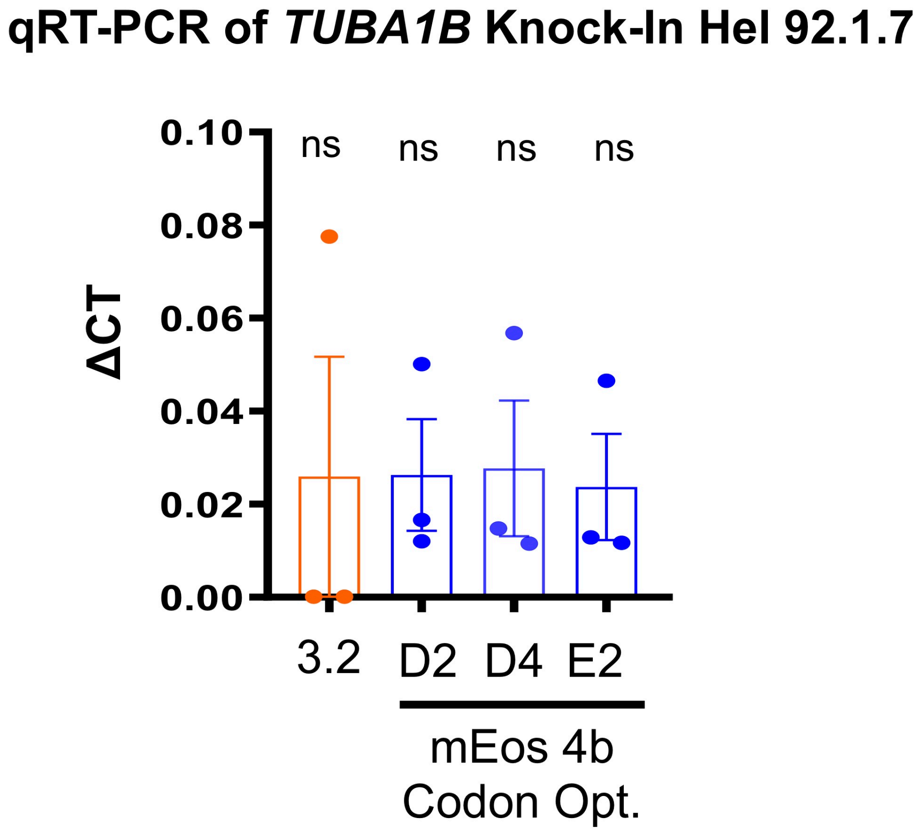

Fig. S7. qRT-PCR comparison of Hek293T knock-in clones. qRT-PCR measurements of mEos 3.2 and mEos 4bCO knock-ins in Hek293T do not show a significant difference in expression between clones at the mRNA level.

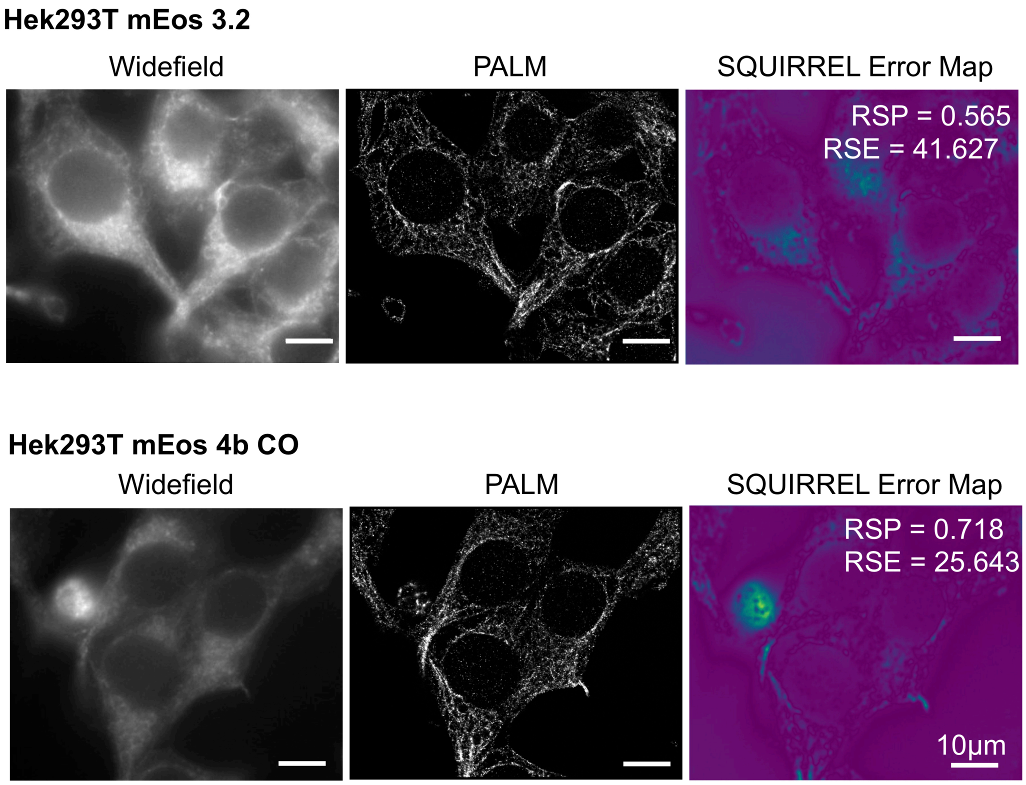

Fig. S8. Quantitative measurement of error in CRISPR-PALM Hek293T knock-in cells. A quantiative comparison of mEos 3.2 and mEos 4b CO clones through SQUIRREL shows an improvement in image quality in mEos 4b CO knock-in clones (RSP 0.565 and 0.718, RSE 41.627 and 25.643 respectively).

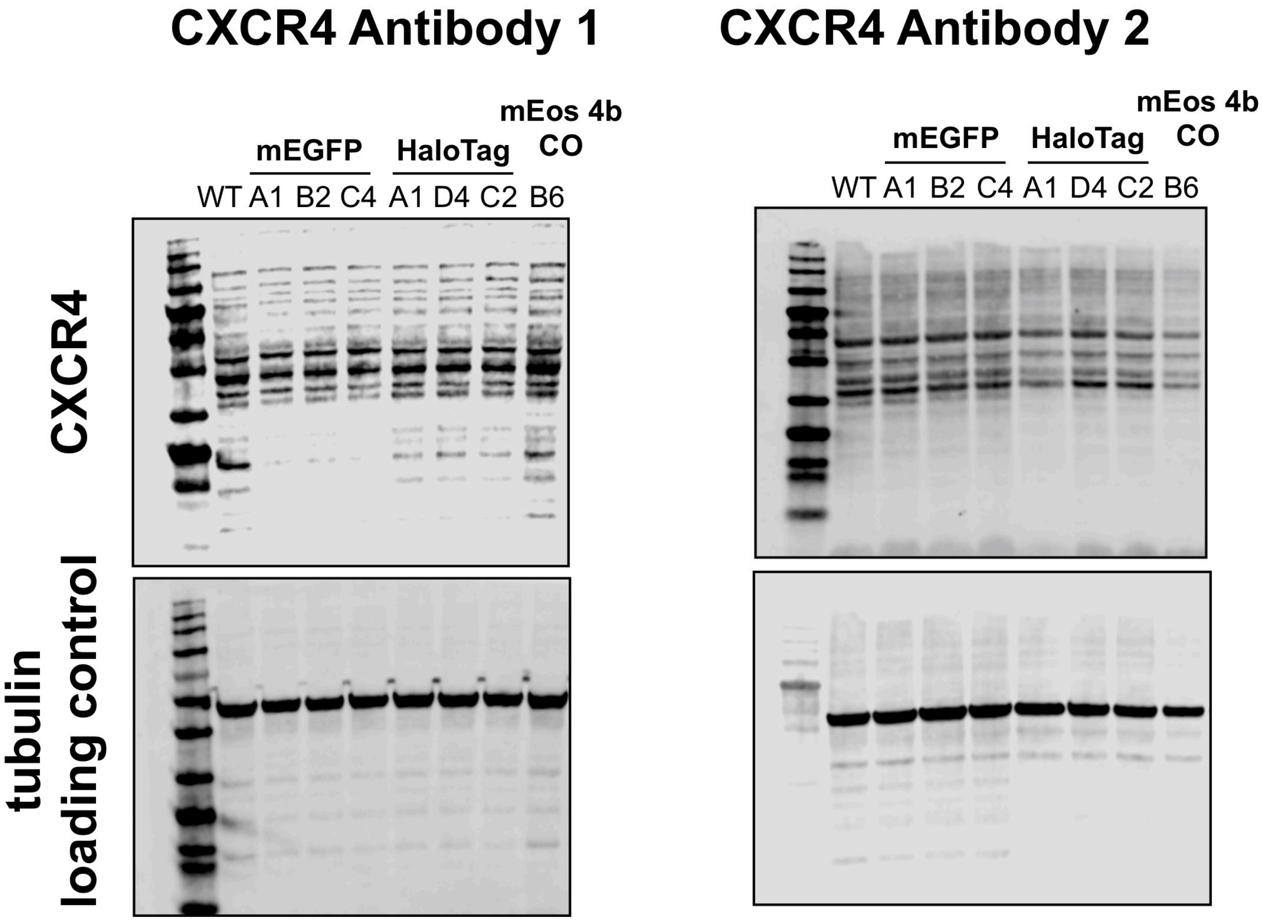

Fig. S9. Western blots of CXCR4 knock-in and wild type Hek293T. Poor detection of CXCR4 with both a knock-out validated and a fusin antibody was observed despite detection and validation by imaging, qRT-PCR, and functional assays.

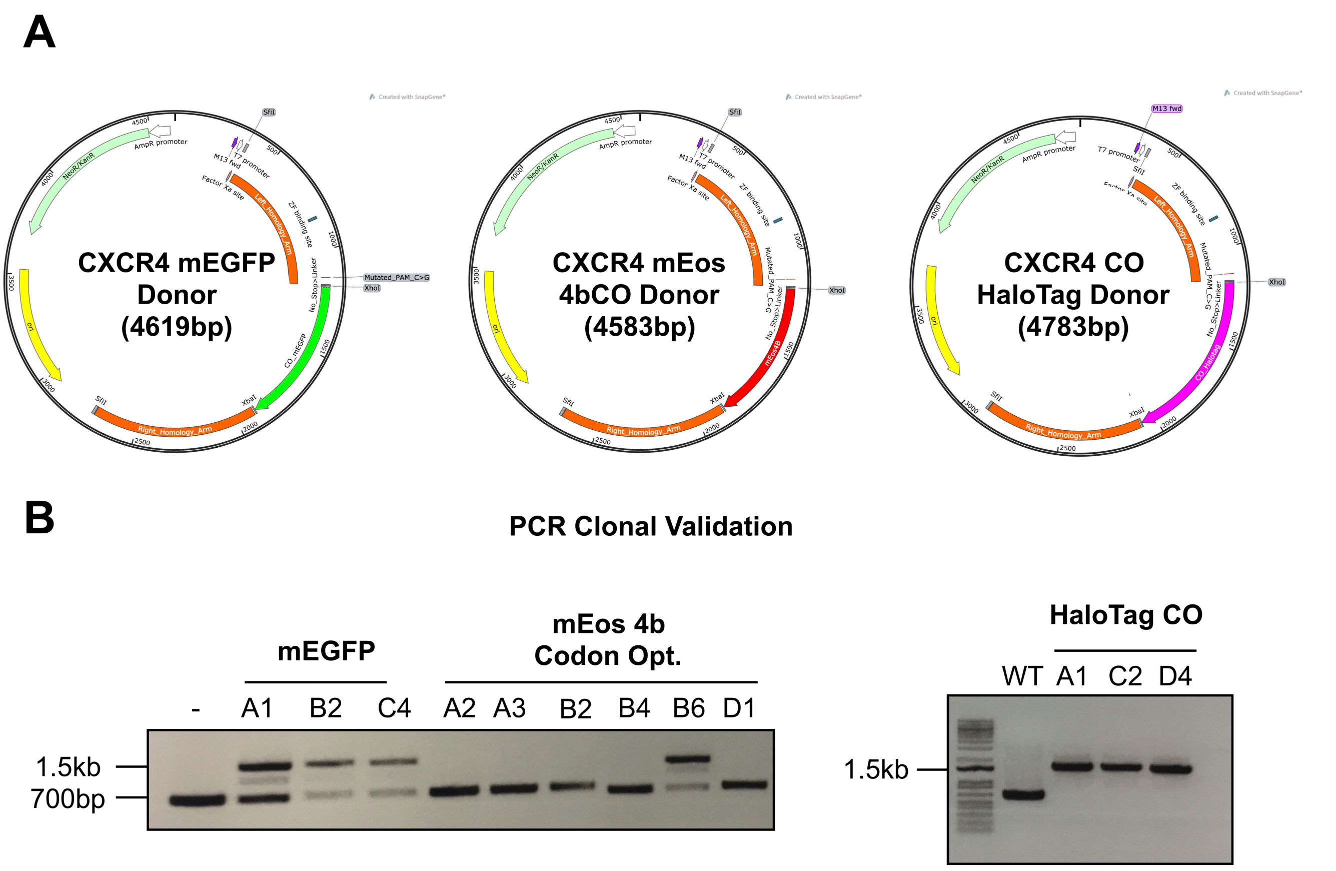

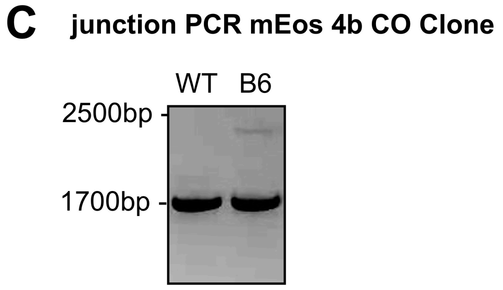

D

CXCR4 Primer Fwd TGTTGGCTGCCTTACTACA
CXCR4 Primer Rev AAACAGCTGGGGATCATTTC

CXCR4 junction Primer GAAGAAACTGAGAAGCATGACG
CXCR4 junction Primer GATGACCTTTCATACAAATAGGTGC

Fig. S10. Design of CXCR4 donor plasmids and genomic validation on knock-in and clonal selection. (A) HDR donor plasmids targeting the CXCR4 C-terminus were generated following the design and cloning strategies previously described by White *et al.*. Donor plasmids carrying mEGFP, mEos 4b CO, and a codon optimised HaloTag sequence were designed and validated by sequencing. (B) On transfection and single cell sorting, cells were validated by PCR to determine the correct insertion of donor sequence at the target loci. mEGFP clones were successfully validated as heterozygous, while mEos 4b CO cells were generally negative due to the low level fluorescence observed (as shown in figure 4, mEos 4b CO appears to agglomerate in the validated clones). HaloTag CO knock-ins proved homozygous for insertion, with a loss of the wild type sequence in the three clones tested. (C) As the mEos 4b CO knock-in appears to misfold, primers flanking donor repair template were designed to confirm correct genomic insertion. For Clone B6, a band at the predicted insertion size 2262bp was observed confirming the CRISPR mediated editing of this gene. (D) Primer sequences for junction and standard validation primers are included here.

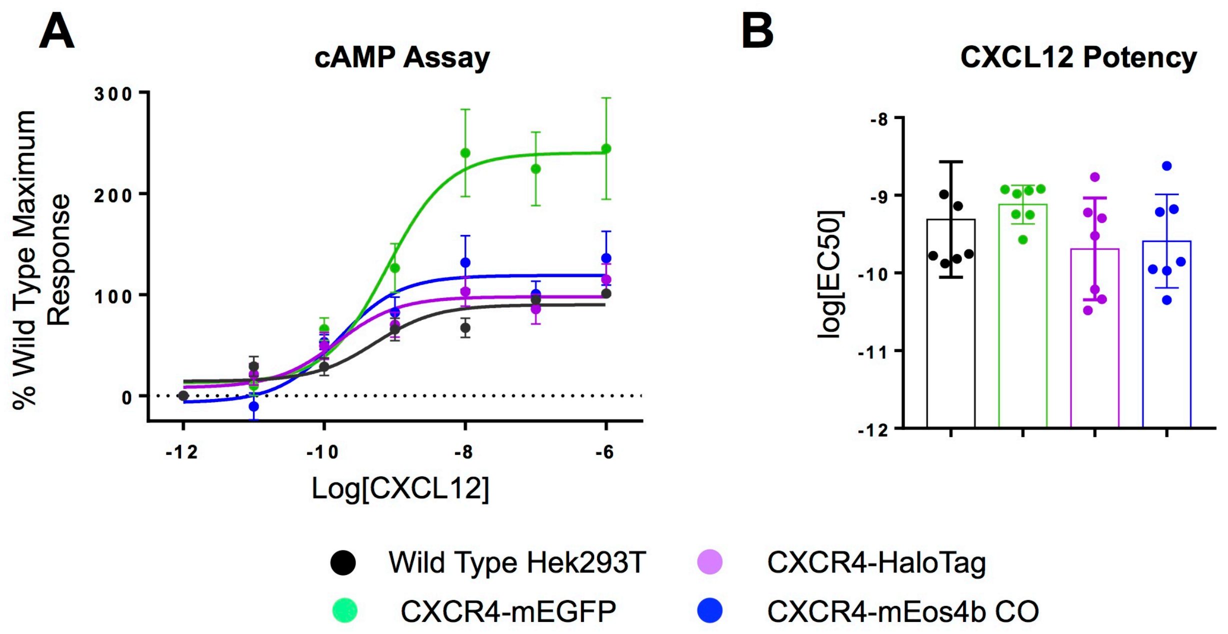

Fig. S11. Functional validation of CXCR4 knock-in clones through cAMP assay. (A) Modulation of cAMP accumulation mediated by CXCL12 (1 pM – 1 μM) in HEK293 cells expressing wildtype or genome-edited CXCR4. (B) Potency of CXCL12-mediated G protein activation in cells expressing wildtype or genome-edited CXCR4. Points and bars represent % maximum CXCL12 response observed in wildtype HEK293 cells $\pm$ S.E.M. of seven independent experiments.

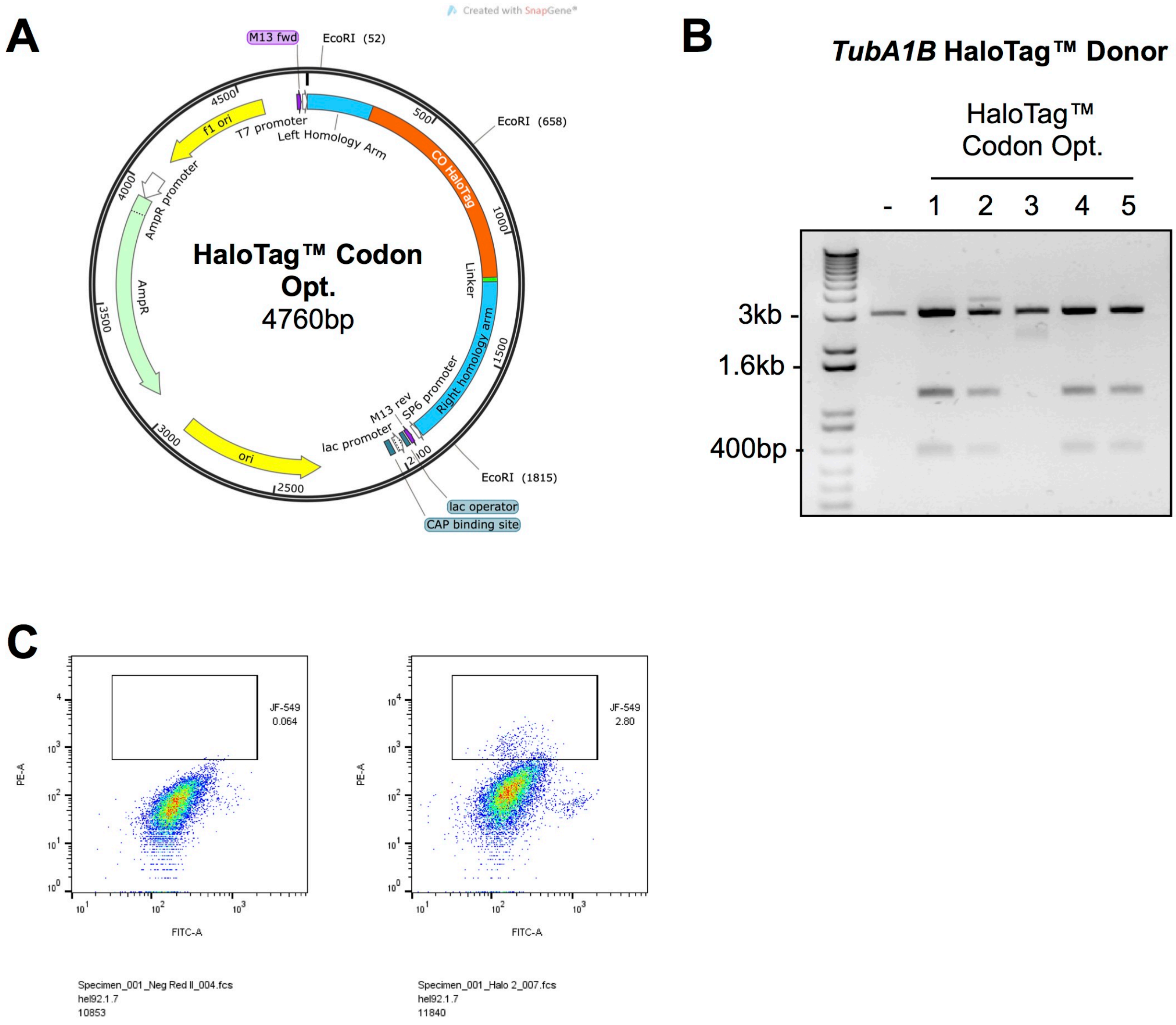

Fig. S12. Generation of *TubA1B* HaloTag CRISPR donor vectors. (A) The *TubA1B* donor template was sub-cloned to include a donor HaloTag sequence as previously described. (B) Vectors were prepped and validated by EcoRI digest before sequencing, with successfully cloned vectors demonstrating two additional bands compared to the negative linearised protein. (C) Hel 92.1.7 cells were co-transfected with the validated HaloTag *TubA1B* donor and a targeting guide, single cell sorting was thenperformed after incubation with Janelia Fluor 549, selecting for the brightest cells as in previous experiments.

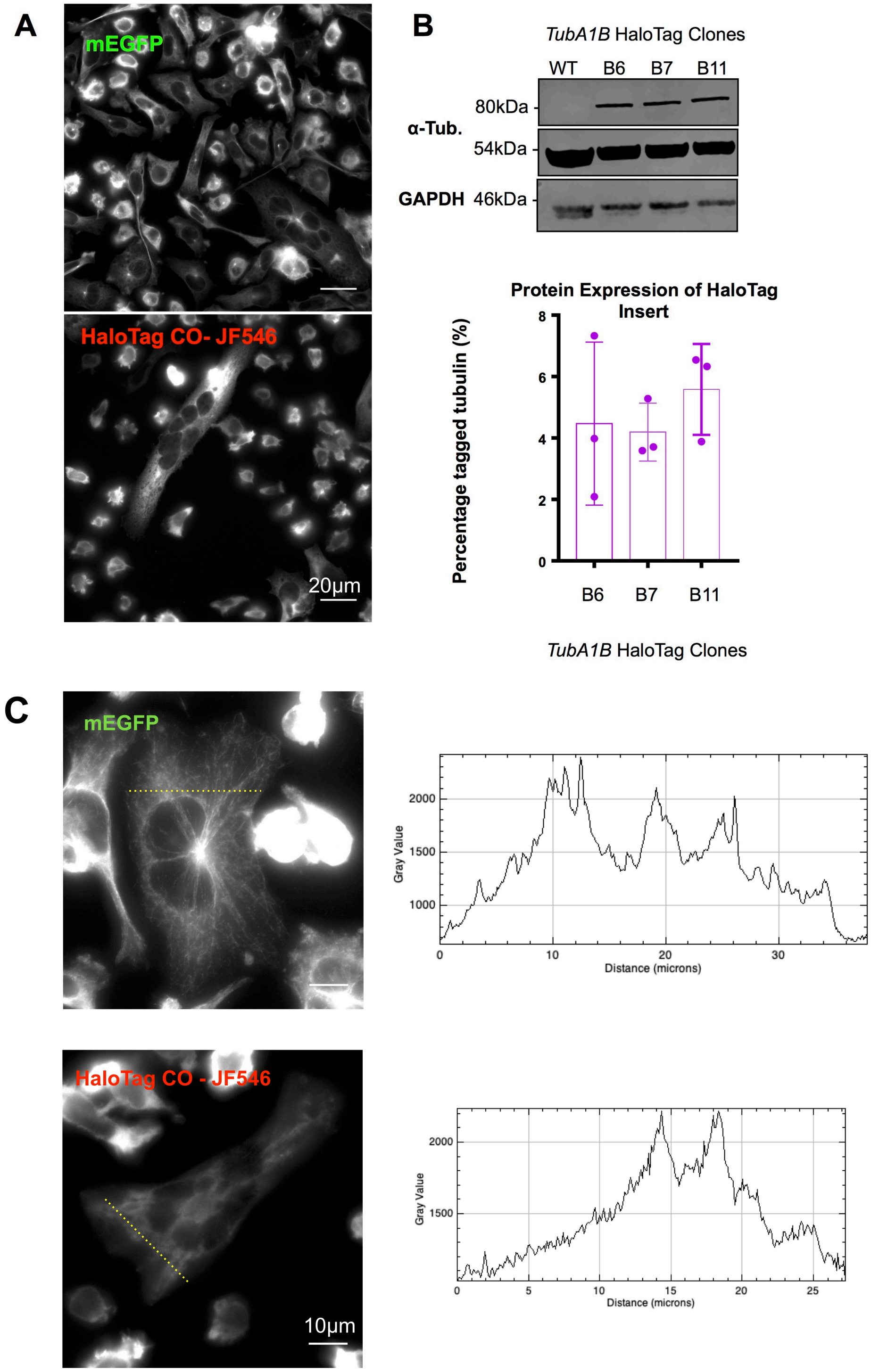

Fig. S13. Validation of HaloTag-*TubA1B* knock-in clones. (A) Hel 92.1.7 cells positive for the HaloTag insert were compared to mEGFP labelled *TubA1B* in the same cell line, while HaloTag labelled cells are fluorescent on incubation with JF549. (B) Quantification of the expression level of HaloTag *TubA1B* shows a substantially reduecd expression of the tagged allele compared to previously reported knock-ins at approximately 5% compared to 20% mEGFP and 18% mEos 4b CO as previously reported. (C) Microtubule labelling is more clearly evident in mEGFP knock-in cells when compared to the HaloTag knock-ins. Similarly, brightness is higher in these cells as shown by the intensity plots.
